# Oxygenation versus driving pressure for determining the best positive end-expiratory pressure in acute respiratory distress syndrome

**DOI:** 10.1186/s13054-022-04084-z

**Published:** 2022-07-13

**Authors:** Saida Rezaiguia-Delclaux, Léo Ren, Aurélie Gruner, Calypso Roman, Thibaut Genty, François Stéphan

**Affiliations:** 1 Cardiothoracic Intensive Care Unit, Hôpital Marie Lannelongue, 133 avenue de la Résistance, 92350 Le Plessis Robinson, France; 2 School of Medicine, Paris-Saclay University, Kremlin-Bicêtre, France; 3 INSERM U999, Pulmonary Hypertension: Pathophysiology and Novel Therapies, Hôpital Marie Lannelongue, Le Plessis-Robinson, France

**Keywords:** Acute respiratory distress syndrome, Positive end-expiratory pressure, Oxygenation, Driving pressure

## Abstract

**Objective:**

The aim of this prospective longitudinal study was to compare driving pressure and absolute PaO_2_/FiO_2_ ratio in determining the best positive end-expiratory pressure (PEEP) level.

**Patients and methods:**

In 122 patients with acute respiratory distress syndrome, PEEP was increased until plateau pressure reached 30 cmH_2_O at constant tidal volume, then decreased at 15-min intervals, to 15, 10, and 5 cmH_2_O. The best PEEP by PaO_2_/FiO_2_ ratio (PEEP_O2_) was defined as the highest PaO_2_/FiO_2_ ratio obtained, and the best PEEP by driving pressure (PEEP_DP_) as the lowest driving pressure. The difference between the best PEEP levels was compared to a non-inferiority margin of 1.5 cmH_2_O.

**Main results:**

The best mean PEEP_O2_ value was 11.9 ± 4.7 cmH_2_O compared to 10.6 ± 4.1 cmH_2_O for the best PEEP_DP_: mean difference = 1.3 cmH_2_O (95% confidence interval [95% CI], 0.4–2.3; one-tailed *P* value, 0.36). Only 46 PEEP levels were the same with the two methods (37.7%; 95% CI 29.6–46.5). PEEP level was ≥ 15 cmH_2_O in 61 (50%) patients with PEEP_O2_ and 39 (32%) patients with PEEP_DP_ (*P* = 0.001).

**Conclusion:**

Depending on the method chosen, the best PEEP level varies. The best PEEP_DP_ level is lower than the best PEEP_O2_ level. Computing driving pressure is simple, faster and less invasive than measuring PaO_2_. However, our results do not demonstrate that one method deserves preference over the other in terms of patient outcome.

*Clinical trial number*: #ACTRN12618000554268. Registered 13 April 2018.

## Introduction

During acute respiratory distress syndrome (ARDS), lung function is similar to a functional baby lung [1]. Optimal ventilatory parameter adjustment is crucial in ARDS [1–3]. A low tidal volume (Vt) has been proven to decrease mortality [4]. However, the optimal level of positive end-expiratory pressure (PEEP) is still matter of controversies [4]. Several methods have been proposed to select the best PEEP level [5, 6], which varied according to the method used. This point may explain the discrepancies across studies comparing high and low PEEP level [2, 7]. Oxygenation based on ARDS Network PEEP/FiO_2_ table [4] or absolute PaO_2_/FiO_2_ ratio is often used to adjust PEEP (PEEP_O2_) [3, 5–11]. The PEEP titration on oxygenation gives different results depending on whether one uses PEEP/FiO_2_ table or the absolute PaO_2_/FiO_2_ ratio.

Driving pressure (DP), computed as plateau pressure (Pplat) minus PEEP, reflects the stress and strain applied to the lung [12]. Lower DP values may be strongly associated with survival [12]. For a given PEEP level, the change in DP is more related to mortality than the change in PaO_2_/FiO_2_ ratio [13]. The best PEEP_DP_ is higher compared to the best PEEP determined by the PEEP/FiO_2_ table [5, 6], but lower when PEEP was determined by absolute PaO_2_/FiO_2_ ratio [14, 15]. A recent study showed that the direction of PEEP change needed to reduce DP was variable from values given in the PEEP/FiO_2_ table [9]. Finally, it is difficult to assume that the PEEP value determined by DP will be higher or lower than that determined by absolute PaO_2_/FiO_2_ ratio.

Our objective here was to compare the value of the best PEEP level using DP (PEEP_DP_) or absolute PaO_2_/FiO_2_ ratio after a decremental PEEP trial.

## Patients and methods

The study protocol was approved by the Ouest IV-Nantes CPP (IRCB # 2018-A01760-55). Informed consent was provided from the patients or relatives. The DROP study (DRiving pressure for Optimization of Positive end-expiratory pressure) was registered on the Australian New Zealand Clinical Trials Registry (#ACTRN12618000554268. Registered 13 April 2018).

Between November 2018 and June 2019, we prospectively included 122 consecutive patients with moderate or severe ARDS as previously defined [16]. Exclusion criteria were age younger than 15 years, chest tube with persistent air leak, and hemodynamic instability.

Patients received neuromuscular blocking agents and volume-controlled ventilation with Vt set at 6 mL/kg of predicted body weight, FiO_2_ at 1, inspiratory/expiratory ratio at 1:2, and respiratory rate at 30/min for a Pplat ≤ 30 cmH_2_O. All patients were in supine position. PEEP was increased until Pplat reached 30 cmH_2_O at constant Vt then decreased at 15-min intervals, to 15, 10, and 5 cmH_2_O. No recruitment maneuver was used and Vt was not reduced during and after PEEP trial. FiO_2_ was set at 1 in order to standardize circumstances [11] and because PaO_2_/FiO_2_ ratio is influenced if FiO_2_ varies greatly. Arterial blood gases were analyzed and lung mechanics recorded after each step. Mean arterial pressure was also collected. The best PEEP_O2_ was defined as a > 10% difference in PaO_2_/FiO_2_ ratio between two consecutive PEEP reduction (this value was defined "a priori" and was based on a previous study [11]), and the PEEP value before this one was considered to be optimal PEEP. The best PEEP_DP_ was defined as the PEEP associated with the lowest DP without knowing the best PEEP_O2_ level.

Continuous variables were described as mean ± SD and compared using Student’s *t*-test if normally distributed and described as median [interquartile range] otherwise. Dichotomous variables were compared by applying the Chi-square test or McNemar’s test. Repeatedly measured quantitative variables were analyzed by ANOVA. To test whether the best mean PEEP_DP_ was not inferior to the best mean PEEP_O2_, with a non-inferiority margin set at 1.5 cmH_2_O and assuming a best PEEP of 10 cmH_2_O with a standard deviation of 3.5 cmH_2_O, 118 patients were required. The margin > 1.5 cmH_2_O was retained because some authors decrease the PEEP level by 2 cmH_2_O during the PEEP trial, which appears to be significant [9, 15].

## Results

**Fig. 1:**
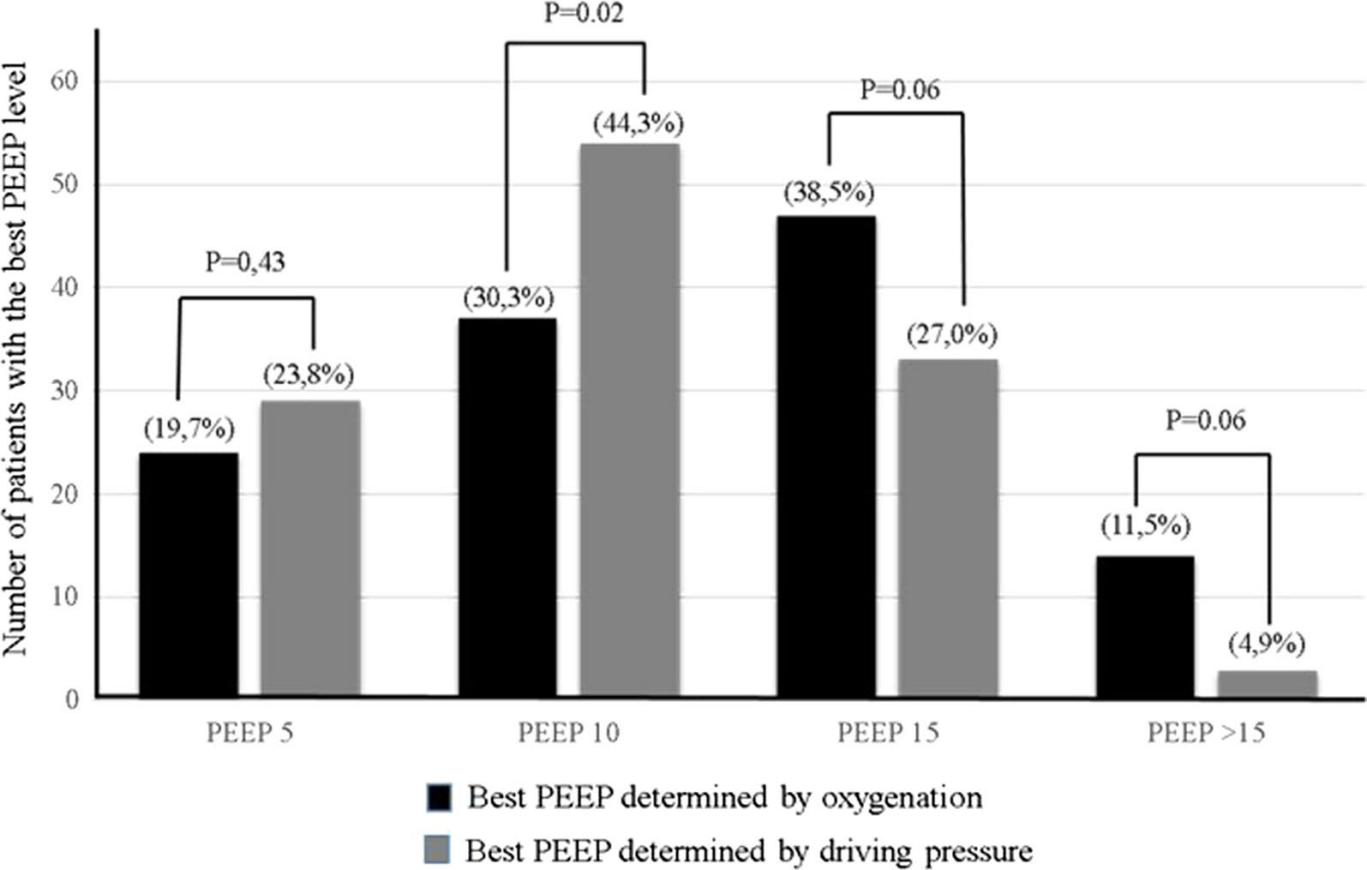
Distribution of the best PEEP levels determined based on absolute PaO_2_/FiO_2_ ratio (PEEP_O2_) or driving pressure (PEEP_DP_). The percentage of patients with each PEEP level differed significantly between the two methods (Chi-2, 9.3; *P* = 0.025)

Table 1 reports the main features of the 122 patients. Table 2 summarizes respiratory mechanics and gas exchanges. Mean arterial pressure did not change significantly across PEEP levels (*P* = 0.71). At a Pplat = 30 cmH_2_O, the PEEP maximal was 17.0 ± 2.3 cmH_2_O. Median auto-PEEP was 1 [IQR 0–1] cmH_2_O. The best mean PEEP_O2_ was 11.9 ± 4.5 cmH_2_O compared to 10.6 ± 4.1 cmH_2_O for the best PEEP_DP_: mean difference = 1.3 cmH_2_O (95% confidence interval [95% CI], 0.4–2.3; one-tailed *P* value, 0.36). Only 46 PEEP levels were the same with the two methods (37.7%; 95% CI 29.6–46.5). The distribution of the best PEEP levels differed significantly between the two methods (*P* = 0.025) (Fig. 1).

**Table 1:** Main features and outcomes of the 122 study patients

Age, years, mean ± SD	58.4 ± 13.8
Males, n (%)	69 (56.5)
Body mass index, mean ± SD	26.5 ± 6.1
Body mass index > 30, n (%)	28 (23%)
SAPSII, mean ± SD	40.2 ± 12.7
Reasons for admission, n (%)	Thromboendarterectomy, 52 (43)
	Lung transplantation, 20 (16)
	Heart surgery, 18 (15)
	Pulmonary resection, 8 (6)
	Heart transplantation, 7 (6)
	Cardiogenic shock, 7 (6)
	Vascular surgery, 4 (3)
	Miscellaneous, 6 (5)
Causes of ARDS, n (%)	Ventilator-associated pneumonia; 50 (41)
	Reperfusion edema/primary graft dysfunction, 49 (40)
	TRALI, 7 (6)
	Septic shock, 6 (5)
	Post-cardiopulmonary bypass, 4 (3)
	Lung graft rejection, 3 (2.5)
	Miscellaneous; 3 (2.5)
Severity of ARDS, n (%)^a^	
Moderate	66 (54)
Severe	56 (46)
Days from admission to PEEP trial, median [IQR]	2 [1–4]
Ventilation parameters before PEEP trial, mean ± SD	
Vt for predicted body weight, mL/kg	5.8 ± 0.7
PEEP level, cmH_2_O	7.9 ± 1.9
Respiratory rate/min, mean ± SD	27 ± 5
PaO_2_/FiO_2_, mmHg, mean ± SD	106 ± 34
Outcomes	
Days on mechanical ventilation, median [IQR]	19 [9–31]
Need for ECMO, n (%)	8 (6.5)
ICU stay length, days, median [IQR]	20.5 [12.0–36.0]
Patients who died, n (%)	20 (16.4)

SAPSII: Simplified Acute Physiology Score version II; ARDS: acute respiratory distress syndrome; TRALI: transfusion-related acute lung injury; PEEP: positive end-expiratory pressure; Vt: tidal volume; PaO_2_/FiO_2_: ratio of partial pressure of oxygen in arterial blood over fraction of inspired oxygen

^a^Moderate ARDS was defined by a PaO_2_/FiO_2_ > 100 mmHg and ≤ 200 mmHg and a PEEP level ≥ 5 cmH_2_O [16]. Severe ARDS was defined by a PaO_2_/FiO_2_ ≤ 100 mmHg and a PEEP level ≥ 5 cmH_2_O [16]

**Table 2:** Respiratory mechanics and gas exchanges according to level of positive end-expiratory pressure in supine position

Variable	PEEP maximal (17.0 ± 2.3 cmH_2_O)	PEEP 15 cmH_2_O	PEEP 10 cmH_2_O	PEEP 5 cmH_2_O	*P* value ANOVA
Driving pressure, cmH_2_O	12.4 ± 2.6	10.6 ± 3.4	9.3 ± 3.0	9.7 ± 3.3	< 0.0001
Plateau pressure, cmH_2_O	30	26.4 ± 3.4	20.2 ± 3.1	15.9 ± 3.5	< 0.0001
Peak pressure, cmH_2_O	45 ± 5	41 ± 6	36 ± 6	33 ± 7	< 0.0001
Respiratory system compliance ml/kg	31.0 ± 11.0	39.1 ± 17.9	44.8 ± 23.7	42.6 ± 20.2	< 0.0001
PaO_2_/FiO_2_, mmHg	188 ± 112	197 ± 106	187 ± 103	153 ± 81	< 0.0001
pH	7.35 ± 0.09	7.35 ± 0.09	7.36 ± 0.09	7.37 ± 0.10	0.01
PaCO_2_, mmHg	46 ± 10	45 ± 10	44 ± 11	44 ± 11	0.01

PEEP: positive end-expiratory pressure; PEEP maximal: the level of PEEP for a plateau pressure = 30 cmH_2_O

Mean differences for PaO_2_/FiO_2_ ratio between best PEEP_O2_ and best PEEP_DP_ were 26.4 [95% CI 17.6–35.0] mmHg (*P* < 0.0001); 1.2 [95% CI 0.9–1.5] cmH_2_O for DP (*P* < 0.0001); 2.4 [95% CI 1.3–3.5] cmH_2_O for Pplat (*P* < 0.0001); and − 6.3 [95% CI − 8.6 to − 3.9] mL/mmHg for respiratory system compliance (*P* < 0.0001). DP was above 14 cmH_2_O in 18 (14.7%) patients with PEEP_O2_ compared to 1 (0.08%) patient by PEEP_DP_ (*P* = 0.06). Pplat was 30 cmH_2_O in 29 (23.8%) patients titrated by PEEP_O2_ compared to 13 (10.6%) patients titrated by PEEP_DP_ (*P* = 0.007).

## Discussion

Using absolute PaO_2_/FiO_2_ ratio or DP in PEEP titration resulted in different best PEEP levels. PEEP level and DP value were higher with PEEP_O2_, which resulted in a larger number of patients having PEEP levels ≥ 15 cmH_2_O. It should be noted that all PEEP titrations were performed in supine position and that these results cannot be extrapolated in prone position [6].

PaO_2_ may imperfectly reflect alveolar recruitment. Thus, alveolar recruitment correlates poorly with oxygenation, as this last results from complex interactions between lung function and hemodynamics [10, 17]. Due to the abnormal behavior of poorly aerated lung tissue, increased recruitment may fail to improve oxygenation [1]. We did not use the PEEP/FiO_2_ table [4] because it does not target the PEEP level to individual lung mechanics. Contrary to previous studies based on PEEP/FiO_2_ table, the best PEEP based on absolute PaO_2_/FiO_2_ ratio was higher than that based on DP as previously reported [5, 6]. In our study, the improvement of PaO_2_/FiO_2_ ratio with increase PEEP was mild. The larger number of patients with PEEP ≥ 15 cmH_2_O when oxygenation method was used raises concern about potential hyperinflation, even when Pplat remains lower than 30 cmH_2_O [7, 18]. Therefore, the oxygenation method may not reliably protect against ventilation-induced lung injury [9, 18]. Thus, some patients were probably at risk for overdistension injury, even while receiving PEEP according to the PEEP/FiO_2_ table [9]. Improved oxygenation after a PEEP increase is barely associated with lower mortality [10, 13].

DP may depend mainly on lung mechanics [12]. Measuring DP at different PEEP levels provides information on the balance between hyperinflation and opening-closing during tidal ventilation [5, 7, 14]. At a constant Vt, PEEP titration based on DP is equivalent to titration based on respiratory system compliance [5, 7, 14]. PEEP increments may be protective only when the increased PEEP values for a same Vt result in a lower DP [12]. In contrast, a DP increase when PEEP is raised indicates a decrease in respiratory compliance, suggesting hyperinflation due to the higher PEEP [7].

Higher survival was observed among patients with lower DP, independent of concomitant variations in PEEP and Pplat [12]. Interestingly, DP is more strongly associated with survival than oxygenation, even after adjustment to Vt [13]. The difference in DP values obtained by PEEP_O2_ or PEEP_DP_ may seem small but it has been suggested that each 1 cmH_2_O increase in DP was associated with a fourfold higher mortality risk [19]. However, oddly enough, the personalized PEEP approach with recruitment maneuver used in the ART trial lowered DP but increased mortality [20].

Our study has limitations. Firstly, the study population was very specific: 60% of patients with ARDS were admitted after thromboendarterectomy and lung transplantation. This does not represent the typical ARDS patients’ category in most ICUs. Secondly, the PEEP effect was tested in the very short term, but DP stabilized within a few minutes of PEEP titration [9]. Thirdly, Vt was left at 6 ml/kg predicted body weight and was not normalized to functional lung size based on lung elastance. However, such approach could minimize bias. Fourthly, we did not use recruitment maneuver, which can influence the PEEP trial, but could also affect mortality [20]. Fifthly, change in DP, as a surrogate for change in transpulmonary pressure, may not be appropriate in patients with extremely low chest wall or abdominal compliance. Finally, the effects of the different PEEP levels on the lung parenchyma were not studied, and we did not record variations of cardiac output.

In conclusion, depending on the method chosen, the best PEEP level varies. The best PEEP level based on DP is lower than that based on oxygenation. Computing DP is simple, faster, and less invasive than measuring PaO_2_. However, despite some previous studies arguing for adjusting the PEEP on DP, our results do not demonstrate that one method deserves preference over the other in terms of patient outcome.

## Data Availability

The datasets used and/or analyzed during the current study are available from the corresponding author on reasonable request.
